# The Role of Lebanon in the COVID-19 Butterfly Effect: The B.1.398 Example

**DOI:** 10.3390/v14081640

**Published:** 2022-07-27

**Authors:** Dalal Nour, Rayane Rafei, Alessandra P. Lamarca, Luiz G. P. de Almeida, Marwan Osman, Mohamad Bachar Ismail, Hassan Mallat, Atika Berry, Gwendolyne Burfin, Quentin Semanas, Laurence Josset, Hamad Hassan, Fouad Dabboussi, Bruno Lina, Philippe Colson, Ana Tereza R. Vasconcelos, Monzer Hamze

**Affiliations:** 1 Laboratoire Microbiologie Santé et Environnement (LMSE), Doctoral School for Science & Technology, Faculty of Public Health, Lebanese University, Tripoli 1300, Lebanon; dalalnour123@gmail.com (D.N.); rayanerafei@hotmail.com (R.R.); marwan.osman@outlook.com (M.O.); bachar.ismail@gmail.com (M.B.I.); hmallat.dr@gmail.com (H.M.); fdaboussi@ul.edu.lb (F.D.); 2 Laboratório de Bioinformática, Laboratório Nacional de Computação Científica, Petrópolis, RJ 25651-075, Brazil; pavanlamarca@gmail.com (A.P.L.); lgonzaga@lncc.br (L.G.P.d.A.); 3 Cornell Atkinson Center for Sustainability, Cornell University, Ithaca, NY 14853, USA; 4 Department of Public and Ecosystem Health, College of Veterinary Medicine, Cornell University, Ithaca, NY 14853, USA; 5 Faculty of Sciences, Lebanese University, Tripoli 1300, Lebanon; 6 Head of the Preventive Medicine Department, Ministry of Public Health, Beirut 1001, Lebanon; aberrymd@gmail.com; 7 Laboratoire de Virologie, Institut des Agents Infectieux, Laboratoire Associé au Centre National de Référence des Virus des Infections Respiratoires, Hospices Civils de Lyon, F-69004 Lyon, France; gwendolyne.burfin@chu-lyon.fr (G.B.); quentin.semanas@chu-lyon.fr (Q.S.); laurence.josset@chu-lyon.fr (L.J.); bruno.lina@chu-lyon.fr (B.L.); 8 GenEPII Sequencing Platform, Institut des Agents Infectieux, Hospices Civils de Lyon, F-69004 Lyon, France; 9 CIRI, Centre International de Recherche en Infectiologie, Team VirPath, Univ. Lyon, Inserm, U1111, Université Claude Bernard Lyon 1, CNRS, UMR5308, ENS de Lyon, F-69007 Lyon, France; 10 Faculty of Public Health, Lebanese University, Beirut 1001, Lebanon; hamad.hassan@ul.edu.lb; 11 IHU Méditerranée Infection, 19-21 boulevard Jean Moulin, 13005 Marseille, France; philippe.colson@univ-amu.fr; 12 Microbes Evolution Phylogeny and Infections (MEPHI), Institut de Recherche pour le Développement (IRD), Aix-Marseille University, 27 boulevard Jean Moulin, 13005 Marseille, France; 13 Assistance Publique-Hôpitaux de Marseille (AP-HM), 264 rue Saint-Pierre, 13005 Marseille, France

**Keywords:** SARS-CoV-2, lineages, B.1.398, Lebanon, dispersal

## Abstract

In the present study, we provide a retrospective genomic surveillance of the SARS-CoV-2 pandemic in Lebanon; we newly sequence the viral genomes of 200 nasopharyngeal samples collected between July 2020 and February 2021 from patients in different regions of Lebanon and from travelers crossing the Lebanese–Syrian border, and we also analyze the Lebanese genomic dataset available at GISAID. Our results show that SARS-CoV-2 infections in Lebanon during this period were shaped by the turnovers of four dominant SARS-CoV-2 lineages, with B.1.398 being the first to thoroughly dominate. Lebanon acted as a dispersal center of B.1.398 to other countries, with intercontinental transmissions being more common than within-continent. Within the country, the district of Tripoli, which was the source of 43% of the total B.1.398 sequences in our study, was identified as being an important source of dispersal in the country. In conclusion, our findings exemplify the butterfly effect, by which a lineage that emerges in a small area can be spread around the world, and highlight the potential role of developing countries in the emergence of new variants.

## 1. Introduction

Since its first diagnosis in December 2019, COVID-19 has been an insurmountable worldwide pandemic, with the number of cases and deaths surpassing 485 million and 6.13 million, respectively, as of 30 March 2022 [1]. The fierce grasp of SARS-CoV-2, the virus that causes COVID-19, is sustained by its ongoing diversification into an enormous number of lineages (1700 recognized by PANGO as of 30 March 2022) [2] bristling with a myriad of new mutations. Some lineages, dubbed the variants of concern (VoCs) Alpha, Beta, Gamma, Delta, and Omicron by the WHO, have gained particular attention due to mutations causing elevated transmissibility, increased disease severity, and impacts on diagnostics and vaccine performance [3]. The resulting increase in the fitness of these VoCs has prompted consecutive lineage turnovers across the world [4,5] and triggered new surges in COVID-19 cases and deaths.

Most of the genomic surveillance of SARS-CoV-2 is conducted in a small number of countries located in North America and Europe. Developing countries originated only ~9.7% of the sequenced genomes available in GISAID (https://www.gisaid.org/ assessed on 9 May 2022) [6,7] despite being home to almost 49% of SARS-CoV-2 cases, as reported by the database on May 9th, 2022 [8]. This may be related to differences across the world in scientific funding, sequencing capacity, and the number of trained researchers [9,10]. The circulation of SARS-CoV-2 under the radar of researchers and public health authorities allows the unnoticed accumulation of worrisome mutations in lineages, resulting in the emergence of VoCs. It is not surprising, then, that most VoCs were first identified in developing countries.

The rapid release and analysis of whole viral genome sequences help researchers to understand the routes of SARS-CoV-2 transmission and paves the way for the implementation of appropriate measures for outbreak reduction and containment. In Lebanon, real-time genome sequencing is not implemented in the SARS-CoV-2 surveillance strategy due to many challenges, including the paucity of sequencing facilities and skilled human staff, the Lebanese economic collapse that exacerbates high sequencing costs, the lack of funding, and reagent delivery delays [11]. Although there were 1,066,840 cases and 10,079 deaths in Lebanon between January 2020 and February 2022 [12,13], only 1199 Lebanese sequences were available in GISAID, which is the world’s largest repository for SARS-CoV-2 genomes as assessed on 4 February 2022 [14].

In our study, we aimed to describe the epidemiological dynamics of SARS-CoV-2 during the first two years of the pandemic in Lebanon. During this period, the average number of recorded Lebanese cases of COVID-19 varied between 5 and 33,605 cases per week [12,13]. We newly sequenced SARS-CoV-2 genomes from 200 nasopharyngeal samples collected between July 2020 and February 2021 from different regions in Lebanon as well as from travelers crossing the Lebanese–Syrian border. Among the lineages identified herein, we selected the first one to thoroughly dominate in Lebanon (B.1.398) to investigate how SARS-CoV-2 transmissions occurred both within Lebanon and between Lebanon and other countries. Finally, we discussed the potential emergence of new variants from developing countries.

## 2. Materials and Methods

### 2.1. Sampling

The study was approved by the ethical committee of the Lebanese University, under the number #CUER 23-2020, on 14 May 2020. The LMSE laboratory (Laboratoire Microbiologie Santé et Environnement) is accredited by the Ministry of Public Health in Lebanon to carry out the diagnosis of SARS-CoV-2 infections in North Lebanon. The diagnoses were carried out by RT-PCR on nasopharyngeal samples using the COVID-19 Genesig Real-time PCR assay (PrimerDesign, Eastleigh SO53 4DG, UK) targeting the RdRp gene; the GeneFinder COVID-19 Plus RealAmp kit (OSANG Healthcare, Dongan-gu, Anyang-si, Gyeonggi-do, Korea) targeting the N, E, and RdRp genes; and the Liferiver Novel Coronavirus (2019-nCoV) Real Time Multiplex RT-PCR kit (Shanghai ZJ Bio-Tech Co, Shanghai, China), which targets the N, E, and ORF1ab genes.

Our samples were collected between July 2020 and February 2021 from several sources: COVID-19 screening campaigns among Lebanese citizens launched by several municipalities in collaboration with the Lebanese Ministry of Public Health, several governmental hospitals in North Lebanon, as well as travelers crossing the Lebanese–Syrian border. All samples positive for SARS-CoV-2 were stored at −80 °C in the LMSE laboratory. The approach adopted for selecting samples for genomic sequencing was, briefly, as follows: all samples with a cycle threshold value (Ct) greater than 25 were excluded to avoid sequencing failure. Then, 200 samples (with Ct ≤ 25) were chosen randomly, respecting the relative proportions of patients of each nationality (the patients were of three nationalities: Lebanese, Syrian, and Palestinian), as well as taking into account the differences between the number of samples in each of the target months. After setting the mentioned parameters and grouping the data in Excel files, we randomized our 200 nasopharyngeal samples taken from patients positive for SARS-CoV-2.

### 2.2. Collecting Information and Handling Samples

Phone calls were made to patients, and after they gave their consent to participate in the study, we filled out a questionnaire gathering socio-demographic information, health data, and data related to SARS-CoV-2 infection (i.e., the source of infection, travel during or before the period of infection, symptoms experienced, whether there was hospitalization or not, and whether or not medication was taken). After obtaining the requested number of participants (200), the corresponding samples underwent a new coding process. Then, total RNAs were extracted at LMSE with the AccuPrep Viral RNA Extraction Kit (BIONEER, Daejeon, Korea) and the QIAamp Viral RNA Kit (QIAGEN, Germantown, TN, USA), according to the recommendations of the suppliers. Finally, the RNA extracts were sent to the Laboratory of Virology, Institute of Infectious Agents, which is associated with the National Reference Center of Respiratory Infection Viruses, Hospice civil de Lyon in Lyon, France, for genomic sequencing by Illumina COVIDSeq (Illumina, San Diego, CA, USA). The amplification of extracted RNA was conducted with an automatized (SPTLabtech) COVIDSeq-Test™ protocol (Illumina) with an ARTIC nCoV-2019 Amplicon Panel v4 of primers. Libraries were sequenced with 100 base pair paired-end reads using the NovaSeq 6000 sequencing system with the SP Reagent Kit v1.0 200 cycles (Illumina) [15]. Reads were processed using the in-house bioinformatic pipeline seqmet, available at https://github.com/genepii/seqmet (accessed on 28 November 2021).

The generation of the consensus sequence and the identification of the SNPs of each genome were performed by aligning the reads with the reference genome (NC_045512.2) using bwa v0.7.17 [16] and post-processing with samtools v1.11, bcftools v1.10.2 [17], and bedtools v2.27.1 [18]. Lineages were identified with the Pangolin program v.3.1.20 [19]. The consensus genomes were added to the GISAID database, and accession IDs are available at https://doi.org/10.55876/gis8.220630un (accessed on 30 June 2022).

### 2.3. Evolutionary and Phylogeographic Analyses

In addition to the genomes sequenced in this study, we obtained the sequences of SARS-CoV-2 genomes collected in Lebanon that were available in the GISAID database as of 7 February 2022, listed at https://doi.org/10.55876/gis8.220630un (accessed on 30 June 2022). This dataset was filtered using the options “Complete”, “Low coverage exclude”, and “Collection date complete” available in GISAID. The final dataset containing the newly sequenced genomes and the GISAID sequences contained 1042 genomes. These sequences were aligned to the WH01 (EPI ISL 406798) genome from Wuhan, China using MAFFT [20]. The 3′ and 5′ ends were trimmed with the seqkit package [21] using the WH01 sequence as a reference. The maximum likelihood tree was then inferred using IQ-TREE [22], using the GTR+F+I+G4 model selected by the built-in algorithm ModelFinder [23] and 1000 ultrafast bootstrap replicates [24].

**Figure A1:**
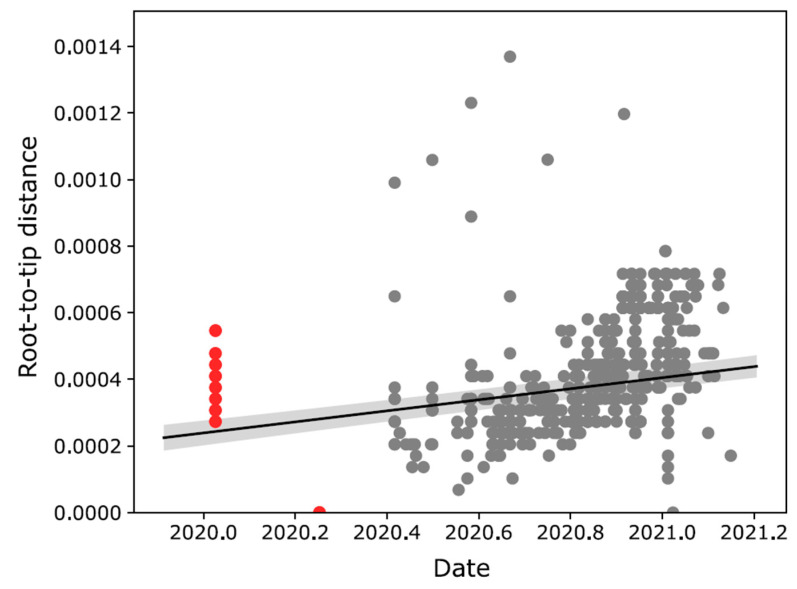
Correlation between sampling dates and the root-to–tip distance of the maximum likelihood tree of lineage B.1.398 when including sequences from GISAID prior to June, 2020 (red points). These sequences were removed for the final analysis.

To evaluate how SARS-CoV-2 has spread across Lebanon, we used the dispersal of the B.1.398 lineage as a case study. To confirm the number of introductions of the lineage in Lebanon, we obtained all sequences of B.1.398 available in GISAID on 7 February 2022, listed at https://doi.org/10.55876/gis8.220630un (accessed on 30 June 2022), using the same filters described previously. Sequences obtained from samples collected before June of 2020 were removed because they had higher diversity than expected based on their sampling dates (Figure A1). Among the excluded sequences were genomes sampled in January 2020, as described in the GISAID database. We believe this date to be an error made during the submission of sequences, as the first confirmed COVID-19 cases in Lebanon occurred in February 2020. We aligned the new B.1.398 genomes and the sequences from GISAID to WH01 with MAFFT, removed the 3′ and 5′ ends with seqkit, and generated a maximum likelihood tree with IQ-TREE. The GTR+F+I substitution model was selected by ModelFinder, and branch support was calculated using 1000 ultrafast bootstrap replicates. The maximum likelihood tree of B.1.398 was used as input to TreeTime [25] to scale the tree branches to divergence dates, assuming the phylogenetic correlation of clock rates (“--covariation” option) and employing a coalescent prior with the skyline model (“--coalescent skyline” option). The ancestral locations of the samples were then inferred using the resulting tree with TreeTime, with the “mugration” model and the countries of origin as the samples’ locations.

The dispersal of B.1.398 within Lebanon was reconstructed using BEAST [26]. Because the sequences from Lebanon available in GISAID did not have information about the district or province of sampling, in this analysis, we used only the sequences generated in this study. We employed the Relaxed Random Walk model with Cauchy’s distribution on coordinates randomly selected within each sample’s collection district; we employed the GTR substitution model with estimated base frequencies and the “Gamma + Invariant Sites” heterogeneity model with four Gamma categories. We also used a coalescent tree prior with the GMRF Bayesian Skyride model and a uniform prior for the clock rate. The MCMC ran through a chain of 100,000,000, with sampling every 10,000th and burn-in of 10% of the trees. Dispersal routes were extracted from the consensus tree using the seraphim package [27] and plotted using the ggplot2 package, both in R software. The base map used in the figures was obtained from https://gadm.org/index.html (accessed on 15 February 2022).

## 3. Results

In the present study, SARS-CoV-2 genomes were sequenced from 200 RNA samples from six of the eight Lebanese provinces (governorates), with the majority of samples being from North Lebanon (70%), followed by Akkar (16%) and Mount Lebanon (11%). The remaining samples were from Bekaa, Nabatieh, and South Lebanon. Among Lebanese districts, the district of Tripoli accounted for the most significant number of samples (36%). To summarize the data, the 200 patients participating in this study were of three nationalities: Lebanese (75.5%), Syrian (17.5%), and Palestinian (7.0%); 54% were male, and 46% were female. Regarding travel status, 10.5% of the patients traveled within 14 days of their infection with SARS-CoV-2: 19 patients to Syria, 1 patient to Italy and Switzerland, and 1 to Turkey. The mean age of the patients was 38.7 years. Regarding infection status, most of the patients (94%) developed symptoms with a mean duration of 10.2 days, and 6% of the patients went to the hospital after infection.

After sequencing, the SARS-CoV-2 genome assembly failed for seven samples, resulting in 193 sequences. We were able to attribute PANGO lineages to all but three of these sequences. In total, 21 PANGO lineages were identified, with B.1.398 predominant (52.5%) followed by the B.1.1.7 lineage (18.5%). With regard to nationalities, the lineages detected in non-Lebanese residents of Lebanon, including Syrians and Palestinians, were generally found in Lebanese citizens as well. Among the travelers to Syria, the lineages were mainly B.1 (36.8%), B.1.1 (21%), and B.1.36 (15.7%). The remaining lineages B.1.1.274, B.1.177, B.1.22, B.1.36.1, and B.1.398 each occurred in 5.3% of Syria travelers. Three of these lineages (B.1.1.274, B.1.177, and B.1.22) were not detected in the Lebanese population. B.1.1.44 was identified in a traveler to Turkey and was not detected in the Lebanese sequences, while B.1.398 was recovered from a traveler from Europe.

**Figure 1:**
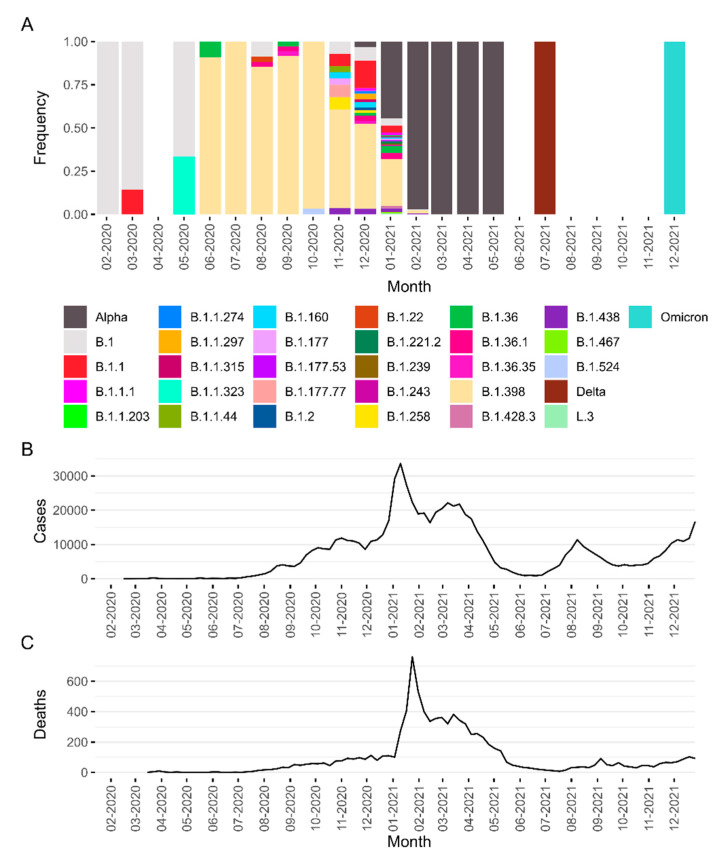
Epidemics of COVID-19 in the first two years of the pandemic in Lebanon. In (**A**), we see the relative frequency of SARS-CoV-2 lineages sampled in Lebanon between February 2020 and December 2021. Lineages belonging to the variants of concern Alpha, Delta, and Omicron are grouped under these labels. The numbers of COVID-19 cases and deaths are described in (**B**,**C**).

Analyzing the whole Lebanese dataset in GISAID, including the new sequences, 40 different lineages were identified within Lebanon for the period ending December 2021. Despite the relatively high diversity in the country during this period, the pandemic in Lebanon can be defined by the dominance of four lineages (Figure 1A). From February 2020 to May 2020, the country repeated worldwide patterns and was dominated by the B.1 lineage. The lineage B.1.398 started to dominate in June of 2020, coinciding with a sharp increase in the number of COVID-19 cases in Lebanon (Figure 1B). The variant of concern Alpha (B.1.1.7 and the sublineage Q.1 in Lebanon) replaced B.1.398 as the predominant lineage in January of 2021, accompanied by a new surge in cases that was more intense than the previous one. Alpha dominated at least until May 2021, the last month of continuous genomic sequencing in Lebanon. The genomes available in GISAID show that by July of 2021, the variant of concern Delta (B.1.617 and the sublineages AY.10, AY.106, AY.120, AY.122, AY.127, AY.16, AY.33, and AY.39 in the country) had already completely substituted Alpha and was, in turn, completely replaced by Omicron (sublineages BA.1 and BA.1.1) by December 2021. Coincidently, the appearances of both lineages (Delta and Omicron) were followed by new increases in the number of cases in Lebanon.

**Figure A2:**
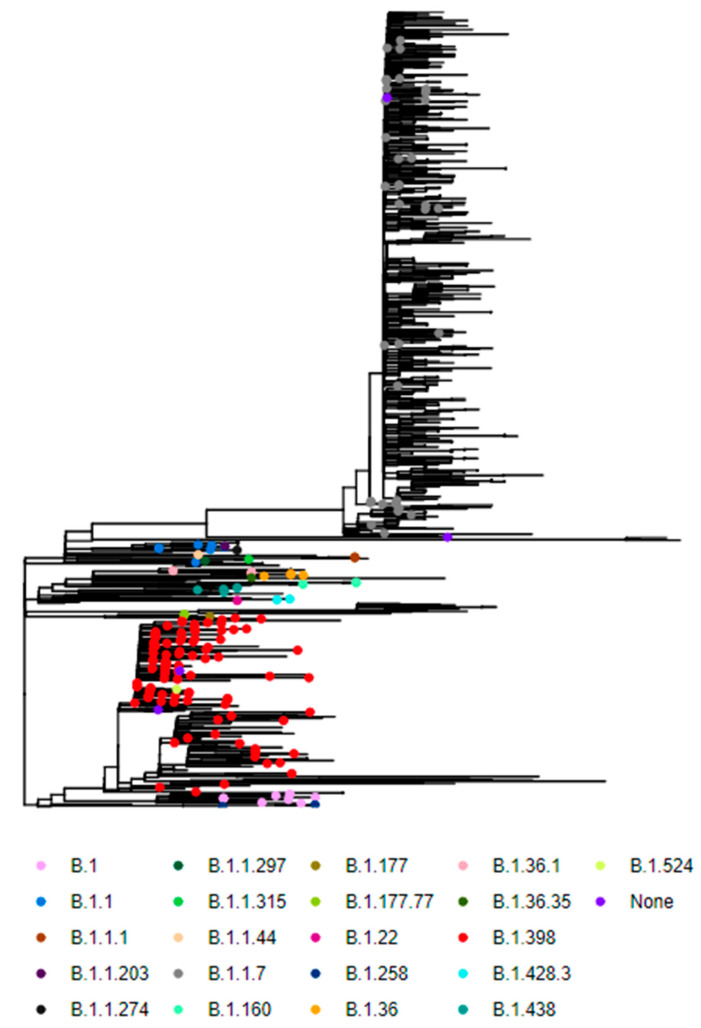
Maximum likelihood phylogeny of SARS-CoV-2 sequences sampled in Lebanon. Colored tip points show the newly-sequenced genomes and their attributed lineage.

To obtain a closer look at the B.1.398 lineage, we performed a double analysis to investigate how and where it was introduced at both the international and national scales. We determined that the lineage originated from B.1 in mid- to late-April 2020, either within Lebanon or in an unsampled country with immediate dispersal to Lebanon. The introduction to Lebanon was followed by a considerable diversification of the lineage, for which many subclades could be delimited. The extent of this heterogeneity was not observed for the Alpha variant, where few subclades could be distinguished, and the majority of sequences were present on a single subclade (Figure A2).

**Figure 2:**
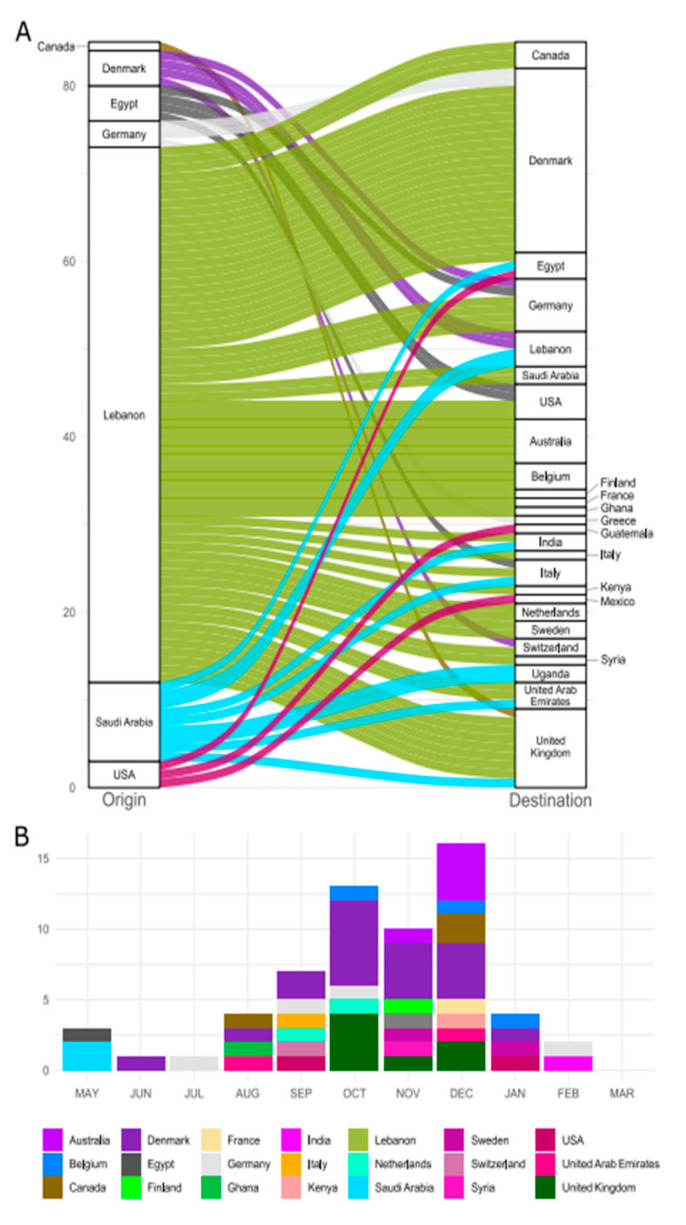
International dispersal of the B.1.398 lineage. In (**A**), all the international transmission routes are represented, with the left bar being the countries of origin of dispersal, the right bar their destinations, and the color of dispersal representing the originating country. In (**B**), the temporal distribution of countries receiving B.1.398 that originated in Lebanon is displayed. The legend for both graphs is located at the bottom.

**Figure A3:**
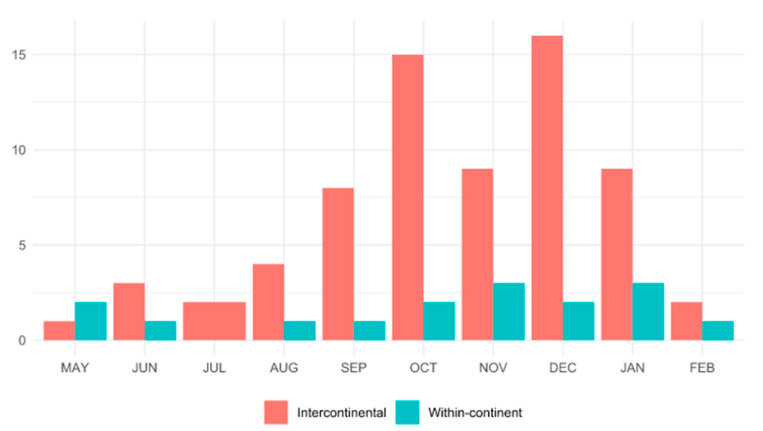
Temporal distribution of intercontinental (red) and within-continent (blue) dispersal of B.1.398 lineage.

By plotting the connections between “origin” and “destination” countries, we see that Lebanon acted as a dispersal center to other countries, with intercontinental transmission being more common than within-continent transmission during the entire period in which the lineage circulated (Figure 2A and Figure A3). The country that received the highest number of importing events of the B.1.398 lineage from Lebanon between June 2020 and January 2021 was Denmark (*n* = 19) (Figure 2B). A high number of transmissions from Lebanon was also identified for the United Kingdom (*n* = 7) between October and December of 2020 and for Saudi Arabia (*n* = 2) in May of 2020. An increase in transmissions of B.1.398 from Lebanon to other countries can be observed beginning in July of 2020, concomitant to the reopening of international flights in the country. Lineage reintroductions into Lebanon from Denmark and Saudi Arabia occurred in January of 2021. These reintroductions did not cause any transmission within Lebanon, as far as the data available show.

**Figure 3:**
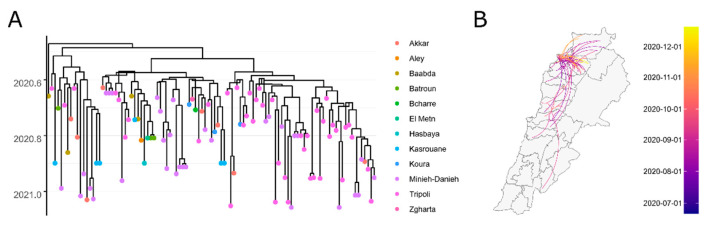
Phylodynamics of the B.1.398 lineage in Lebanon. In (**A**), we see the time tree of B.1.398, indicating divergence times between genomic sequences, with colors representing the districts in Lebanon. The Tripoli district acted as a dispersal hub in the country, as displayed in (**B**). Colors in (**B**) indicate the time at which dispersal started.

Within the country, we identified that the district of Tripoli, which was the source of 43% of the total B.1.398 sequences, acted as an important source of dispersal in the country, especially as the origin of long-distance transmissions (Figure 3). The first long-distance dispersals happened between Tripoli and the district of Akkar, while in a later stage of the pandemic, dispersals were occurring between Tripoli and the southern and northern districts in similar proportions.

## 4. Discussion

In the present study, we provided a retrospective genomic surveillance of the main SARS-CoV-2 lineages circulating between July 2020 and February 2021 in Lebanon by sequencing a set of 200 samples and analyzing the Lebanese genomic dataset available at GISAID as of 7 February 2022. We then focused our analysis on the B.1.398 lineage by reconstructing the transmission routes on both Lebanese and worldwide scales. The new genomes sequenced in this work more than doubled the number of sequences available for the first year of the pandemic in Lebanon. In particular, it greatly improved the sampling of the B.1.398 lineage in this period (Figure A2), which is critical for understanding how SARS-CoV-2 first spread across Lebanon and caused the first surge of COVID-19 cases in the country.

In comparison with previous studies [28,29,30], seven variants not available among GISAID’s Lebanese sequences (B.1.1.203, B.1.1.274, B.1.1.44, B.1.177.77, B.1.22, B.1.428.3, and B.1.524), were detected in our study. Among them, B.1.1.44 was detected in a patient returning from travel to Turkey, and B.1.1.274 and B.1.22 were detected in patients returning from Syria within 14 days of infection. On the other hand, unlike the study by Merhi et al. [30], our results showed that the Alpha variant was detected in Akkar province, with a percentage of 6.8% of our sequences.

Moreover, our findings revealed that the turnover of many SARS-CoV-2 lineages shaped the epidemic in Lebanon. The first officially confirmed COVID-19 case was signaled on 21 February 2020. Although the number of sequences is limited, there was apparently transmission of the B.1 lineage from February to May 2020, triggered by its first introduction from a patient returning from the city of Qom, Iran [29]. The B.1 lineage was an important European lineage originating from the Northern Italian outbreak that occurred early in 2020 [31]. During this period, the number of weekly confirmed Lebanese cases was low, reaching a maximum of 195 cases; this was associated with a strict nationwide lockdown [12,13]. In June 2020, although the international airport was closed, a new dominant lineage, namely B.1.398, was detected. This lineage was characterized by the mutation V1291I in the ORF1a gene, P314L in ORF1b, D614G and T95I in the spike gene, S194L in the nucleocapsid gene, and S84L in ORF8. The D614G mutation in the spike protein gene has been suggested as being responsible for an evolutionary advantage in comparison to the lineages that first circulated in Wuhan [32,33,34]. The presence of this lineage might be explained either by unnoticeable circulation during the first few months due to the low number of sequences available from the initial period, which is a factor we discussed herein, or by recent introduction (or emergence) from an unknown source. Unfortunately, there are no sequences from April in the Lebanese GISAID dataset to support this hypothesis. Although our samples are not equally distributed across the different Lebanese districts, and the district of Tripoli represented the biggest number of samples (66 without travelers), the dispersal analysis of the B.1.398 lineage uncovers the magnitude of transmissions occurring between the districts regardless of their geographical closeness. Community spread appeared to be promoted by many seeding events in which Tripoli may have played a central role. Indeed, Tripoli is the second-largest city in Lebanon, located 82 km from the capital Beirut, and it is the largest city in North Lebanon [35]. Additionally, in June, Lebanon witnessed many anti-government protests and the easing of the COVID-19 lockdown [36], which may explain the lineage’s spread. Still, the number of cases remained low, with a maximum of 183 weekly confirmed cases [12,13]. We previously suggested that the emergence of the lineage might have been related to the airport opening in July 2020 (1148 weekly confirmed cases at maximum) and/or the massive Beirut blast on 4 August 2020 (4084 weekly confirmed cases at maximum during this month) [12,13]. However, the airport opening in July did not seem to introduce this lineage into Lebanon but appears to have played an important role in disseminating it to the world (Figure A3). Despite the absence of new sequences from Beirut, the massive blast that hit the city, with 6500 people injured, 220 deaths, and 300,000 displaced, had rippling consequences on the status of COVID-19 in Lebanon [37]. Indeed, the blast fragmented the healthcare sector, leading to severe damage to Beirut’s major hospitals and overwhelming the remaining functional hospitals with blast casualties. Many injured individuals were transported to nearby regions, and many Lebanese across all provinces rushed to volunteer to clear the rubble, distribute food, etc. [38]. This intermingling served as a breeding ground for SARS-CoV-2 to disseminate, culminating in a new COVID-19 wave in which the chief lineage was presumably B.1.398, as emphasized by the BEAST time tree and dispersal route analysis. The preponderance of this lineage over others remained discernible until December despite the imposition of a lockdown until November 2020 [39]. During its prevalence period (June to December), the number of weekly cases varied between 94 and 16,936, and weekly deaths varied between 0 and 109 [12,13]. Commensurate with the dynamic shifts in lineages observed worldwide [40], “Alpha variant was reported for the first time in Lebanon at the end of December 2020, and swept other lineages in the next following months. This quantum leap for the Alpha variant was potentially energized by the holiday months, specifically, December and January, which aligned with the removal of lockdown restrictions; January was the month with the highest daily morbidities and mortalities since the beginning of the pandemic [1,12,13]. Therefore, a full lockdown was set for 15 January 2021. Despite the reintroduction of the B.1.398 lineage in January from outside the country, its transmission faded and could not be sustained, suggesting thus a lower adaptive value than that of the Alpha variant. Such a disappearance could also be tied to the introduction of the vaccine to Lebanon on 14 February 2021 [41]; the B.1.398 lineage had potentially lower immune evasion than the Alpha variant and there was probably higher vaccination efficacy against it. If we compare cases and deaths in the period in which B.1.398 was prevalent (i.e., between June and December 2020: 189,865 cases, 1378 deaths) and the period in which the Alpha variant was prevalent (between January and May 2021: 355,015 cases, 6282 deaths), we observe the higher transmissibility and mortality of the Alpha variant [12,13].

To our knowledge, there are no sufficient data available in the literature regarding the B.1.398 lineage. According to the PANGO lineages website, this lineage appeared approximately in January 2020 and was denoted as a European lineage [2]. Before our study, there were 377 sequences of this lineage in the GISAID dataset, derived from 24 countries; almost all sequences were from Denmark 138 (37%), Lebanon 116 (31%), the United Kingdom 24 (6%), Germany 19 (5%), and Saudi Arabia 16 (4%) [14]. In our study, we added to GISAID 105 new B.1.398 sequences from Lebanon, of which one sequence is from a traveler crossing the Lebanese–Syrian border. Although this lineage did not encounter eminent success compared to the variant of concerns, we also demonstrated herein the ability of Lebanon to spread and seed B.1.398 to different continents worldwide. While Lebanon is a small country, with a population estimated at more than six million, it has a prodigious number of expatriates (between 4 and 13 million) scattered around the world; this fact explains the extent of connections observed here during holidays [42]. The geographic location of Lebanon also renders it a vivid hub for travel to and from all over the world. In addition to the Lebanese population, Lebanon provides shelter for many refugees, including Syrians and Palestinians, making Lebanon the tenth most populated country with respect to refugees; it also has the highest per-capita concentration of refugees in the world [43,44], allowing Lebanon to be an area of swift COVID-19 transmission and the emergence of potential new SARS-CoV-2 variants. This phenomenon may embody the butterfly effect, where a lineage that emerged in a small area can propagate around the world.

Moreover, Lebanon has witnessed many scourges that converged with and exacerbated the COVID-19 pandemic. Most important was the catastrophic economic and financial crisis that ranked among the world’s three worst crises since the mid-1800s; it caused more than 80% of the Lebanese population to sink into poverty and created shortages of essential medicines, fuel, and electricity. Therefore, the government was not able to apply anti-COVID-19 measures with a tight rein, and for some Lebanese, COVID-19 was the least of their concerns [45,46].

It is worth mentioning that our conclusions may be limited by the absence of historical data both in Lebanon and in neighboring countries. In Lebanon, there is a substantial lack of sequences on both the temporal and spatial scales as many months and districts have no (or a limited number of) sequences. In Syria, there is a lack of genomic surveillance, and only 89 Syrian sequences obtained in Turkey from a short period (December 2021 and January/February 2022) are available in the GISAID database (6 April 2022); these samples correspond to 10 lineages, with BA.1.1 and AY.122 being the most prevalent. We demonstrated here a diverse pool of lineages harbored by the travelers crossing the Lebanese–Syrian border, of which three lineages, B.1.1.274, B.1.177, and B.1.22, were not detected in the Lebanese population, hinting at the possible existence of a specific Syrian pool. With regard to the identified lineage B.1.398, our analysis showed that Lebanon spread this lineage to Syria. However, these conclusions could be altered if more samples from Syria were analyzed.

Furthermore, the current analysis cannot determine with certainty whether dispersal from Lebanon to other countries occurred directly or through stepping stones due to the sampling/sequencing bias caused by the inequality of genomic surveillance across the world. This inequality may also be the reason that the data show dispersal from Lebanon to European countries being more common than to close countries.

Therefore, fostering real-time genomic surveillance in developing countries is a cornerstone of monitoring and tracking the spread and evolution of the virus. These countries constitute potential hot spots for the emergence of new variants because they are regions where SARS-CoV-2 transmissibility is high, and there is an elevated likelihood of multi-variant coinfection and recombination, combined with low levels of surveillance, intervention, and sequencing capacities. Examining a series of retrospective sequences has allowed us, in this work, to bring into sharp focus the importance of small and interconnected countries such as Lebanon in sowing such variants as the B.1.398 lineage around the world. Therefore, the earlier genomic surveillance is implemented, the earlier the first seeding events will be unraveled, and the stronger the worldwide response against new variants will be.

## Data Availability

All the accession numbers of the genomes used in this study are presented in https://doi.org/10.55876/gis8.220630un (accessed on 30 June 2022).

## References

[1] Our World in Data. https://ourworldindata.org/explorers/coronavirus-data-explorer.

[2] Pango Lineages: Latest Epidemiological Lineages of SARS-CoV-2. https://cov-lineages.org/.

[3] WHO: Tracking SARS-CoV-2 Variants. https://www.who.int/en/activities/tracking-SARS-CoV-2-variants/.

[4] Brussow H. (2021). COVID-19: Emergence and mutational diversification of SARS-CoV-2. Microb. Biotechnol..

[5] Dubey A., Choudhary S., Kumar P., Tomar S. (2021). Emerging SARS-CoV-2 Variants: Genetic Variability and Clinical Implications. Curr. Microbiol..

[6] World Economic Situation and Prospects Country Classification. https://www.un.org/en/development/desa/policy/wesp/wesp_current/2014wesp_country_classification.pdf.

[7] Elbe S., Buckland-Merrett G. (2017). Data, disease and diplomacy: GISAID’s innovative contribution to global health. Glob. Chall..

[8] GISAID. https://www.gisaid.org/submission-tracker-global/.

[9] Chen Z., Azman A.S., Chen X., Zou J., Tian Y., Sun R., Xu X., Wu Y., Lu W., Ge S. (2022). Global landscape of SARS-CoV-2 genomic surveillance and data sharing. Nat. Genet..

[10] Brito A.F., Semenova E., Dudas G., Hassler G.W., Kalinich C.C., Kraemer M.U.G., Ho J., Tegally H., Githinji G., Agoti C.N. (2021). Global disparities in SARS-CoV-2 genomic surveillance. medRxiv.

[11] Koweyes J., Salloum T., Haidar S., Merhi G., Tokajian S. (2021). COVID-19 Pandemic in Lebanon: One Year Later, What Have We Learnt?. mSystems.

[12] WHO Health Emergency Dashboard. https://covid19.who.int/region/emro/country/lb.

[13] Republic of Lebanon Ministry of Public Health (MOPH) Epidemiological Surveillance Program. Official Updates Coronavirus-COVID-19 In Lebanon. https://www.moph.gov.lb/en/.

[14] GISAID. https://www.gisaid.org/.

[15] Bal A., Simon B., Destras G., Chalvignac R., Semanas Q., Oblette A., Queromes G., Fanget R., Regue H., Morfin F. (2022). Detection and prevalence of SARS-CoV-2 co-infections during the Omicron variant circulation, France, December 2021—February 2022. medRxiv.

[16] Li H., Durbin R. (2009). Fast and accurate short read alignment with Burrows-Wheeler transform. Bioinformatics.

[17] Danecek P., Bonfield J.K., Liddle J., Marshall J., Ohan V., Pollard M.O., Whitwham A., Keane T., McCarthy S.A., Davies R.M. (2021). Twelve years of SAMtools and BCFtools. Gigascience.

[18] Quinlan A.R., Hall I.M. (2010). BEDTools: A flexible suite of utilities for comparing genomic features. Bioinformatics.

[19] Rambaut A., Holmes E.C., O’Toole A., Hill V., McCrone J.T., Ruis C., du Plessis L., Pybus O.G. (2020). A dynamic nomenclature proposal for SARS-CoV-2 lineages to assist genomic epidemiology. Nat. Microbiol..

[20] Katoh K., Standley D.M. (2013). MAFFT multiple sequence alignment software version 7: Improvements in performance and usability. Mol. Biol. Evol..

[21] Shen W., Le S., Li Y., Hu F. (2016). SeqKit: A Cross-Platform and Ultrafast Toolkit for FASTA/Q File Manipulation. PLoS ONE.

[22] Minh B.Q., Schmidt H.A., Chernomor O., Schrempf D., Woodhams M.D., von Haeseler A., Lanfear R. (2020). IQ-TREE 2: New Models and Efficient Methods for Phylogenetic Inference in the Genomic Era. Mol. Biol. Evol..

[23] Kalyaanamoorthy S., Minh B.Q., Wong T.K.F., von Haeseler A., Jermiin L.S. (2017). ModelFinder: Fast model selection for accurate phylogenetic estimates. Nat. Methods.

[24] Hoang D.T., Chernomor O., von Haeseler A., Minh B.Q., Vinh L.S. (2018). UFBoot2: Improving the Ultrafast Bootstrap Approximation. Mol. Biol. Evol..

[25] Sagulenko P., Puller V., Neher R.A. (2018). TreeTime: Maximum-likelihood phylodynamic analysis. Virus Evol..

[26] Suchard M.A., Lemey P., Baele G., Ayres D.L., Drummond A.J., Rambaut A. (2018). Bayesian phylogenetic and phylodynamic data integration using BEAST 1.10. Virus Evol..

[27] Dellicour S., Rose R., Faria N.R., Lemey P., Pybus O.G. (2016). SERAPHIM: Studying environmental rasters and phylogenetically informed movements. Bioinformatics.

[28] Fayad N., Abi Habib W., Kandeil A., El-Shesheny R., Kamel M.N., Mourad Y., Mokhbat J., Kayali G., Goldstein J., Abdallah J. (2021). SARS-CoV-2 Variants in Lebanon: Evolution and Current Situation. Biology.

[29] Feghali R., Merhi G., Kwasiborski A., Hourdel V., Ghosn N., Tokajian S. (2021). Genomic characterization and phylogenetic analysis of the first SARS-CoV-2 variants introduced in Lebanon. PeerJ.

[30] Merhi G.T.A., Martins L., Koweyes J., Le-Viet T., Abou Naja H., Al Buaini M., Prosolek S., Alikhan N.F., Lott M., Tohmeh T. (2021). Replacement of the Alpha variant of SARS-CoV-2 by the Delta variant in Lebanon between April and June 2021. MedRxiv.

[31] Lineage List. https://cov-lineages.org/lineage_list.html.

[32] Jackson C.B., Zhang L., Farzan M., Choe H. (2021). Functional importance of the D614G mutation in the SARS-CoV-2 spike protein. Biochem. Biophys Res. Commun..

[33] Isabel S., Grana-Miraglia L., Gutierrez J.M., Bundalovic-Torma C., Groves H.E., Isabel M.R., Eshaghi A., Patel S.N., Gubbay J.B., Poutanen T. (2020). Evolutionary and structural analyses of SARS-CoV-2 D614G spike protein mutation now documented worldwide. Sci. Rep..

[34] Plante J.A., Liu Y., Liu J., Xia H., Johnson B.A., Lokugamage K.G., Zhang X., Muruato A.E., Zou J., Fontes-Garfias C.R. (2021). Spike mutation D614G alters SARS-CoV-2 fitness. Nature.

[35] Medcities. https://medcities.org/fr/member/tripoli/.

[36] Hubbard B., Saad H. (2020). Lebanon’s Currency Plunges, and Protesters Surge Into Streets. The New York Times.

[37] Cheaito M.A., Al-Hajj S. (2020). A Brief Report on the Beirut Port Explosion. Mediterr. J. Emerg. Med. Acute Care.

[38] Cheeseman A. (2020). After Beirut Explosion, Lebanese Volunteers Flock to Help Clean up. NBC News.

[39] Aboud C. (2020). Lebanon: A New General Quarantine Begins to Counter the Rise in Corona Virus Infections (Available in Arabic Version). France24.

[40] Gomez C.E., Perdiguero B., Esteban M. (2021). Emerging SARS-CoV-2 Variants and Impact in Global Vaccination Programs against SARS-CoV-2/COVID-19. Vaccines.

[41] Mumtaz G.R., El-Jardali F., Jabbour M., Harb A., Abu-Raddad L.J., Makhoul M. (2021). Modeling the Impact of COVID-19 Vaccination in Lebanon: A Call to Speed-Up Vaccine Roll Out. Vaccines.

[42] Tabar P., Denison A. (2020). IMISCOE Research Series. Diaspora Policies, Consular Services and Social Protection for Lebanese Citizens Abroad. Migr. Soc. Prot. Eur. Beyond.

[43] World Population Review. https://worldpopulationreview.com/countries/lebanon-population.

[44] International Labor Organization. https://www.ilo.org/global/programmes-and-projects/prospects/countries/lebanon/lang--en/index.htm.

[45] Unated Nations ESCWA. https://www.unescwa.org/publications/multidimensional-poverty-lebanon-2019-2021.

[46] Hubbard B. (2021). As Lebanon’s Crisis Deepens, Lines for Fuel Grow, and Food and Medicine Are Scarce. New York Times.

